# Celiac disease in children and adolescents at a singe center in Saudi Arabia

**DOI:** 10.4103/0256-4947.75779

**Published:** 2011

**Authors:** Omar I. Saadah

**Affiliations:** From the Department of Pediatrics, Faculty of Medicine, King Abdulaziz University Hospital, King Abdulaziz University, Jeddah, Saudi Arabia

## Abstract

**BACKGROUND AND OBJECTIVES:**

Celiac disease (CD) is an immune-mediated enteropathy, induced by gluten in genetically susceptible individuals. The objective of this study was to describe the clinical pattern of CD in children from the western region of Saudi Arabia.

**DESIGN AND SETTING:**

Retrospective, hospital-based.

**PATIENTS AND METHODS:**

This study included children with a biopsy-proven diagnosis of CD made between September 2002 and July 2007. Children were admitted to the endoscopy unit for a small-bowel biopsy if they had gastrointestinal symptoms suggestive of CD or if they were positive for a CD-antibody screen performed for the high-risk groups.

**RESULTS:**

Eighty children were identified with a diagnosis of CD. Their mean (SD) age was 9.6 (4.9) years (range, 0.5-18 years). There were 44 (55%) female patients. Forty-one (51%) patients were detected during screening of high-risk groups, while 39 (49%) patients had classical symptoms of malabsorption. The screening also detected asymptomatic patients. Of 65 patients tested, 11 (17%) had elevated liver function tests, which reverted to normal after introduction of a gluten-free diet (GFD) except in one case. Seventy-three (91%) patients were positive for anti-tissue transglutaminase antibodies, 18 (23%), for IgG anti-gliadin antibodies; and 46 (58%), for IgA anti-gliadin antibodies. Forty-one (56%) patients showed good adherence to GFD as assessed by dietary history and the decline in anti-tTG level.

**CONCLUSION:**

CD may present with classical symptoms or be identified through screening programs. Growth and laboratory abnormalities usually improve after introduction of a GFD. Adherence to a GFD remains a problem; therefore, thorough assessment and counseling at the time of diagnosis and ongoing care are crucial.

Celiac disease (CD) is an immune-mediated enteropathy, caused by a permanent sensitivity to ingested gluten in genetically susceptible individuals. The disorder is common, occurring in 0.5% to 1% of the general population in most European countries.^1^ In the past, CD was thought to exclusively affect people of European origin. New, simple, very sensitive and specific serological tests have now become available, and these have shown that CD is common, not only in Europe, but also in developing countries where the major staple diet is wheat.^2^ In developing countries, both serological screening in the general population and serological testing in groups at risk are necessary for early identification of CD patients. Reports of a high prevalence of CD in Egypt^3^ and Tunisia^4^ indicate that the disease is also common in the Arab population.

There are no reported national epidemiological studies of mass screening for CD in children in Saudi Arabia. However, Al Attas^5^ has reported a seroprevalence for CD of 7.6% in a reference laboratory setting among the 145 patients with clinically suspected disease and 2.5% among 18 patients with various autoimmune diseases; none of her patients with inflammatory bowel disease or healthy blood donors were seropositive for CD.

Implementation of a gluten-free diet (GFD) poses a challenging public health problem in developing countries such as Saudi Arabia, since commercial gluten-free products are not widely available. The diagnosis can be obtained through demonstration of the characteristic histological changes (including villous atrophy) on small intestinal biopsy and the resolution of the mucosal lesions and symptoms upon withdrawal of gluten-containing foods.^5^ CD may present with classical symptoms of malabsorption, such as chronic diarrhea, abdominal distension and growth failure, or it can be identified through screening of high-risk groups.^6^,^7^

The aim of this retrospective study was to describe the clinical picture, anthropometric changes and laboratory abnormalities of a group of children diagnosed with CD and to discuss the challenges faced in management, namely, adherence to GFD and the availability of commercial GFD products.

## METHODS

We identified retrospectively all patients who had been diagnosed with CD at King Abdulaziz University Hospital, Jeddah, Saudi Arabia, in the period between September 2002 and July 2007. Children were admitted to the endoscopy unit for a small-bowel biopsy if they had gastrointestinal symptoms suggestive of CD or if they were positive for a CD-antibody screen performed for the high-risk groups. Small bowel biopsy specimens were obtained by upper gastrointestinal endoscopy performed by the author. Two to four specimens from the distal duodenum were sent for histopathology. The diagnosis of CD was based on compatible serologic tests, small bowel biopsy and response to a GFD. At the time of diagnosis, all patients received education about a GFD. Patients attended the gastroenterology clinic every 4 months for follow-up. Serial measurements of weight, height, triceps skin fold thickness and mid-arm circumference were obtained immediately before the diagnosis of CD and during the clinic visits in the first 12 months after the introduction of GFD. The z scores for ‘weight for age’ and ‘height for age’ were calculated by using an anthropometric software program (EpiInfo, Centers for Disease Control and Prevention, Atlanta, GA, USA). During the follow-up visits, dietary awareness and adherence to GFD were assessed by taking a detailed dietary history from older children and/or their guardians. Measurements of celiac-specific antibodies (tissue transglutaminase or anti-gliadin antibodies) were done at 6 and 12 months after beginning of a GFD. A decrease of more than 50% in the antibody titer, with eventual disappearance in most children, is taken as an indirect indicator of dietary adherence and recovery.^8^

Statistical analysis was performed using Stata Statistical Software (Stata Corporation, Release 6.0, College Station, TX, USA). Results are expressed as percentage of the total, or as median with interquartile range. Repeated-measure ANOVA on rank was performed to test for changes during the follow-up period. Statistical significance was accepted if P value was less than .05.

## RESULTS

Eighty children were identified with a diagnosis of CD. Their mean (SD) age at diagnosis was 9.6 (4.9) years (range, 0.5-18 years). There were 44 (55%) female patients. Forty-one (51%) patients were detected during screening of high-risk individuals, while 39 (49%) had presented with classical symptoms of malabsorption. The characteristics of each group are presented in **Table 1**. The clinical presentation of the 39 children classified in the classical group included chronic diarrhea in 32 (82%), anorexia in 32 (82%), abdominal distension in 30 (77%), poor weight gain in 30 (77%), and abdominal pain in 12 (31%) patients. One patient presented at the age of 8 years with the rare presentation of celiac crisis, which included severe diarrhea and dehydration associated with metabolic acidosis, hypokalemia, hypocalcaemia, hypomagnesemia and hypoproteinemia.

**Table 1:** Characteristics of screening-detected and classical-symptoms patients with celiac disease.

	Screening-detected CD n=41	Classical CD n=39	*P*
Age (mean [SD]) in years	9.7 (4.2)	9.4 (5.6)	
Male: female ratio	21:20	15:24	
Saudis/non-Saudis	22/19	19/20	
Nutritional status			
Weight for age z score (mean [SD])	–1.44 (1.2)	–1.5 (1.9)	.9
Height for age z score (mean [SD])	–1.69 (1.3)	–1.5 (1.35)	.5
BMI z score (mean±SD)	–0.69 (1.28)	–0.68 (1.3)	.9
Skin fold thickness (cm)	4 (1.09)	3.4 (1.1)	.15
Mid-arm circumference (cm)	20 (3.7)	20 (4.5)	.8
Albumin (g/L)	37.6 (4.4)	32.8 (8.4)	.002
Hemoglobin (g/dL)	11.48 (1.7)	10 (1.8)	.001
Calcium (mmol/L)	2.2 (0.37)	2.1 (0.37)	.7
Phosphate (mmol/L)	1.5 (0.28)	1.47 (0.37)	.8
Alkaline phosphatase (IU)	350 (275)	295 (174)	.34

BMI: Body mass index

Forty-one patients were detected during screening of high-risk groups. Twenty-three (56%) patients were identified during routine screening of children with type 1 diabetes mellitus (DM). The mean age at diagnosis was 9 (4.2) years (range, 2-18 years), and there were 11 (48%) female patients. Four had occasional abdominal pain and 1 patient had diarrhea. Another 14 (35%) patients were identified during screening of children with isolated short stature; 9 (64%) were females. The mean age at diagnosis was 11.2 (4.5) years (range, 3-16 years), with mean ‘height for age’ z score at diagnosis of –3.07±1. Only two of them reported occasional nonspecific abdominal pain. Four (10%) additional patients were identified during screening of the families of affected individuals.

In this series, CD was associated with other disorders, including autoimmune disorders, as well as other disorders of nonimmune origin (**Table 2**). Two patients presented with seizure disorders; of these, one was a 16-year-old boy who had a history of repeated seizures for 1 year prior to the diagnosis of CD and was treated with sodium valproate, but without control. His brain CT scan was unremarkable, with no occipital calcifications. The other patient was an 18-year-old girl with history of repeated seizures for 5 years prior to the diagnosis, treated with topiramate with some improvement. Her CT scan was unremarkable, but her MRI brain showed a scattered high signal intensity in both cerebral hemispheres interpreted as subcortical edema, post-epilepsy status. Both patients had better seizure control following the diagnosis of CD and institution of GFD.

**Table 2:** Associated comorbidity in 80 patients with celiac disease at diagnosis.

	Number	Percentage
Autoimmune disorders		
Type 1 DM	23	29
Autoimmune thyroiditis	6	7
Autoimmune hepatitis	1	1
Systemic lupus erythematosus	1	1
Vitiligo	1	1
Other disorders		
Osteomalacia	3	4
Seizure disorders	2	2
Down syndrome	2	2
IgA deficiency	1	1

### 

#### Anthropometric parameters

**Figure 1:**
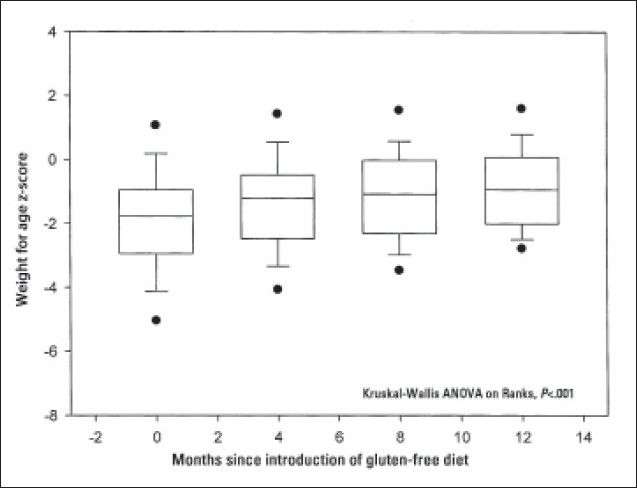
Weight changes in 80 patients with celiac disease following the introduction of a gluten-free diet. Dots are oulying values. Floor and ceiling of box are 25th and 75th percentile. Center line is median.

**Figure 2:**
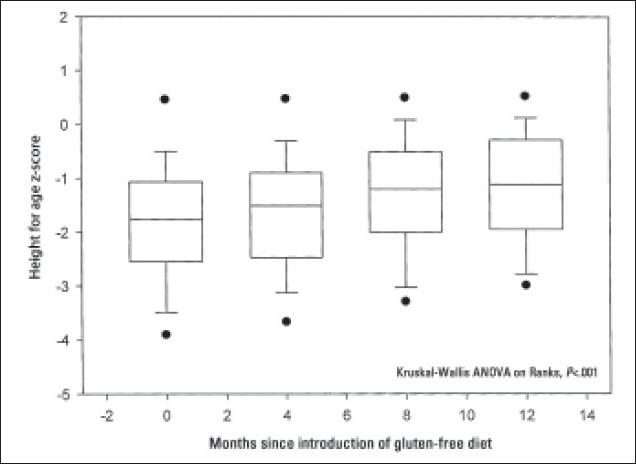
Height changes in 80 patients with celiac disease following the introduction of a gluten-free diet. Dots are oulying values. Floor and ceiling of box are 25th and 75th percentile. Center line is median.

**Figure 3:**
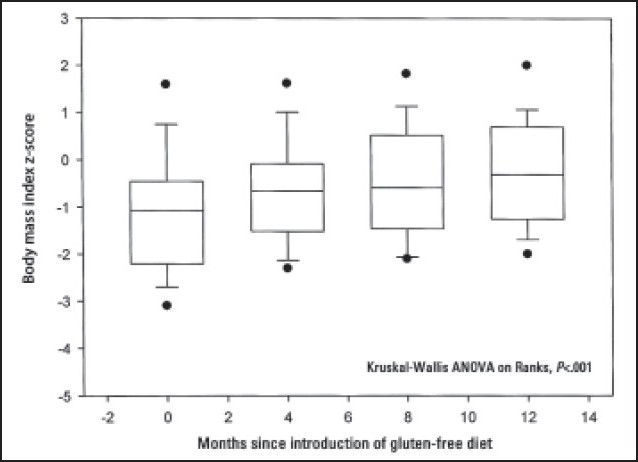
Changes in body mass index following the introduction of a gluten-free diet in 80 children with celiac disease. Dots are oulying values. Floor and ceiling of box are 25th and 75th percentile. Center line is median.

Twenty-eight (35%) patients had weight below the third percentile at presentation, with the mean ‘weight for age’ z score (SD) of –3 (1.3) (range, –5.8 to –2.2). Thirty (38%) patients had short stature with height below the third percentile and mean ‘height for age’ z score (SD) of –2.9 (0.75) (range, –4.7 to –2.11). Eighteen (23%) patients had body mass index z score less than –2 with the mean (SD) of –2.35 (0.25) (range, –2.7 to –2). Following the introduction of a GFD, serial measurements of weight, height and body mass index for the whole group showed significant improvement over a period of 12 months (ANOVA, *P*<.001) (**Figures 1–3**). Nutritional status as indicated by serial measurements of subcutaneous fat thickness and triceps mid-arm circumference was also found to be improved after the introduction of a GFD for the whole group (ANOVA, *P*<.001). In subgroup analysis according to adherence, patients with good adherence to the GFD demonstrated significant difference when compared with the non-adherent group in the respective mean values of weight for age z scores, BMI and skin fold thickness (ANOVA, *P*<.001) but not in the respective mean values of height for age z scores and triceps mid-arm circumference (ANOVA, *P*=.58 and ANOVA, *P*=.84, respectively).

**Table 3:** Biochemical changes during the 12-month follow-up period after introduction of a gluten-free diet (n= 80).

Months	0	4	8	12	*P*
	Median P (25%, 75%)	Median (25%, 75%)	Median (25%, 75%)	Median (25%, 75%)	
Hemoglobin (g/dL)	11.8 (10, 12.8)	12.5 (10.75, 13)	12.85 (11.6, 13.5)	13.3 (11.97, 13.9)	<.001
Albumin (g/L)	35 (34, 38)	37.5 (36, 40)	38.5 (37, 41)	40.5 (39, 43)	<.001
Calcium (mmol/L)	2.23 (2.15, 2.29)	2.2 (2.14, 2.28)	2.23 (2.16, 2.28)	2.19 (2.14, 2.22)	.09
Phosphate (mmol/L)	1.46 (1.3, 1.6)	1.44 (1.3, 1.6)	1.45 (1.3, 1.6)	1.41 (1.2, 1.6)	.4
Alkaline phosphatase (IU)	229 (140, 298)	233 (137, 305)	213 (124, 294)	193 (133, 275)	.2

#### Laboratory parameters

At presentation, albumin level was low (less than 35 g/L) in 28 (35%) patients, with a mean (SD) value of 27.6 (6) g/L (range, 14 to 34 g/L). Fifty-three (66%) patients were anemic with a hemoglobin less than 12 g/dL with a mean (SD) of 9.8 (1.4) g/dL (range, 4.2 to 11.9 g/dL). Only 3 patients had corrected calcium level less than 2.2 mmol/L, with a mean value of 1.94 mmol/L. Repeated measurements of hemoglobin and albumin levels during the 12-month follow-up period showed significant improvement following the introduction of GFD (ANOVA, *P*<.001); however, repeated measurements of calcium, phosphate and alkaline phosphatase showed no significant changes (ANOVA, *P*=.09, 0.4, 0.2, respectively) (**Table 3**).

Measurements of liver enzymes alanine aminotransferase (ALT) and aspartate aminotransferase (AST) were performed in 65 patients at diagnosis. Eleven (17%) patients had elevated ALT and AST levels, with a mean (SD) ALT of 115 (95) IU and a mean (SD) AST of 118 (196) IU. Following the introduction of a GFD, the levels of both ALT and AST were normalized in 10 patients. One patient failed to respond to GFD and required corticosteroids after being subsequently confirmed as an autoimmune hepatitis (type 1) patient.

#### Serological testing for celiac-related antibodies

Serological testing was done in all 80 patients at diagnosis. Most patients had more than one serological testing performed. Seventy-three (91%) patients had positive anti-tissue transglutaminase antibodies (anti-tTG) at diagnosis (more than 20 units), with a mean (SD) value of 118 (84) units. IgG anti-gliadin antibodies were positive (more than 20 units) in 18 (23%) patients with a mean (SD) value of 125 (102) units, while IgA anti-gliadin antibodies were positive in 46 (58%) patients with a mean (SD) value of 74 (71) units. Seven (9%) patients were not tested for anti-tTG and tested positive for IgA anti-gliadin antibodies.

#### Histopathology

The initial small-bowel biopsy in all patients showed severe enteropathy. All except 1 (98%) patient had total or subtotal villous atrophy with either completely flat villi or with clearly atrophic but recognizable villi. Only 1 patient had partial villous atrophy, in which the villi were blunt and shortened. Villous atrophy in all patients was associated with compensatory crypt hyperplasia and increased prominence of intraepithelial lymphocytes.

#### Introduction of GFD and assessment of adherence

GFD was started in all patients at diagnosis. Adherence to GFD was followed using detailed dietary history and repeated measurements of anti-tTG, which were readily available for follow-up as per hospital policy, to gradually replace anti-gliadin antibodies. Adherence was tested only in the 73 patients who were positive for anti-tTG at diagnosis. Forty-one (56%) patients showed significant decline in their initial level of anti-tTG during the first 6 months following introduction of GFD. The correlation between adherence as judged by the detailed dietary history and as assessed by the decline of anti-tTG titer was strong and significant (r= 0.8, *P*=.001). This reflects a very good agreement between assessment of adherence as judged by careful dietary history and the assessment done using serology.

## DISCUSSION

Celiac disease is an immunologically mediated enteropathy caused by exposure to ingested gluten in genetically susceptible individuals.^1^ This results in significant damage to the small intestinal mucosa and malabsorption. Children with CD used to present with classical symptoms of chronic diarrhea and malabsorption, especially in the era before the introduction of serological testing. Since the introduction and development of more sensitive and specific serological tests, the spectrum of symptoms has changed. Children with CD may not present with chronic diarrhea, but rather be identified during the presentation with nonclassical symptoms, or during the routine screening of high-risk individuals, like children with type 1 DM and isolated short stature.

More than half of the patients described in this study were identified during screening of the high-risk individuals, including children with type 1 DM and children with isolated short stature; or as part of family screening. Most of those patients had no history of chronic diarrhea or symptoms of malabsorption. This finding is different from what was reported by Assiri et al,^9^ where diarrhea was the main presentation in 34 (57%) patients out of 62 Saudi children described. The higher proportion of children with diarrhea may be related to the younger age at diagnosis reported in their study of 6.5 years, since classical CD tends to present more in the younger age group. Patients with asymptomatic CD, however, are still at risk of complications such as autoimmunity^10^,^11^ and small-bowel malignancies.^12^

The development of serological markers for CD contributed enormously to the diagnosis of CD in children, especially children presenting with nonclassical symptoms.^7^ The most recently introduced test of anti-tTG was the most specific and sensitive test for the diagnosis of CD.^13^ A significant proportion of children with CD can be detected by screening children with genetic risk, such as children with type 1 DM. Type 1 DM has been associated with diabetes mellitus,^14^ and a higher percentage was reported from our region.^15^,^16^ It was considered the most common indication for regular screening for CD. Both conditions share a common genetic background. Twenty-three (29%) patients in this series were diabetics. Most of them were discovered during routine screening for CD performed at our diabetic clinic. Children with CD and type 1 DM could benefit from introducing a GFD, which improves their diabetic control and stimulates their growth, as previously reported.^17^

Because an autoimmune mechanism has been associated with CD, other autoimmune diseases may coexist with CD. In this series, apart from type 1 DM, other autoimmune disorders were identified, such as autoimmune thyroid disease in 7% of patients. Also autoimmune hepatitis, systemic lupus erythematosus and vitiligo were diagnosed. Each was present in 1% of the patients. Longstanding undiagnosed CD is believed to be the cause behind emergence of other autoimmune diseases.^11^

Growth failure was considered one of the sole manifestations of CD.^18^ It could result from nutrient malabsorption or reduced intake due to anorexia.^19^ Twenty-eight (35%) patients were underweight and 30 (38%) patients had short stature. Significant improvement in growth parameters was observed following the introduction of GFD, indicating improvement in intestinal absorption due to healing of the damaged intestinal mucosa. This growth improvement was associated with improvement in muscle bulk and subcutaneous fat as measured by mid-arm circumference and skin fold thickness, respectively.

Furthermore, 53 (66%) patients were anemic and 28 (35%) patients had low albumin. The presence of anemia and low albumin was more pronounced in the classical group than the screening-detected group (**Table 1**). This indicates that malabsorption of nutrients may be the main underlying cause; however, nutritional deficiencies alone may not explain this phenomenon in all cases.^20^ Recovery is usually possible with GFD alone.^21^ The loss of albumin through the damaged mucosa may contribute to the low serum albumin observed in such patients. The level of albumin usually improves following mucosal recovery.

The two main forms of liver damage (cryptogenic and autoimmune) appear to be related to CD.^22^ The GFD normalizes the cryptogenic form, but most likely not the autoimmune hepatitis.^23^ Nonalcoholic fatty infiltration of the liver causing steatohepatitis may be associated with elevated liver enzymes in CD patients.^24^,^25^ In this series, 65 patients were tested for abnormal liver enzymes. ALT and AST were elevated in 11 (17%) patients. Ten patients responded to a GFD with normalization of both ALT and AST levels. One patient did not respond to GFD alone and was further tested for the possibility of autoimmune hepatitis. He tested strongly positive for both ANA and anti–smooth muscle antibodies. This patient responded dramatically to corticosteroid treatment with eventual normalization of his liver enzymes. None of the patients in this study had evidence of fatty infiltration of the liver on ultrasound examination.

The severity of intestinal mucosal damage was graded by Marsh^26^,^27^ from I to III. All patients but one reported in this series had subtotal and total villous atrophy, representing the severe form of enteropathy, which corresponds to Marsh IIIB and IIIC, respectively. Only 1 patient had partial villous atrophy corresponding to Marsh IIIA. The severe grades of villous atrophy in the studied patients may reflect the long duration of gluten exposure prior to the diagnosis and the possible delay in seeking medical care.

The cornerstone of treatment for CD is elimination of gluten from the diet. In most patients diagnosed with CD, a strict GFD alone results in complete resolution of symptoms and histological recovery, with subsequent reduction in the risk of complications. Noncompliance with GFD is the leading cause of failure of response in patients with CD. The reported adherence rate to GFD in children and adolescents in the Western literature was 56% to 81%.^28^–^30^ These variations may be due to different methods used to assess adherence in different studies. In this study, the combination of dietary history and the repeated measures of serological testing (anti-tTG) was used to assess adherence, as previously reported.^8^,^31^ Forty-one (56%) patients demonstrated a decline in the antibody level during the first 6 months of follow-up.

The noncompliance rate of 44% in this series was high as a greater proportion of our patients were older children and adolescents. This indicates that the adherence rate in this study was comparable to that in similar reports from Europe and North America. The lack of availability of commercial GFD products was an obstacle shared by many children in this study. They did not have alternatives to substitute for the food items they liked but had to rely only on exclusion of gluten-containing food. The lack of availability of GFD products could adversely influence the rate of adherence and compliance.

In conclusion, a significant proportion of children with CD may present with nonclassical symptoms. Most have severe mucosal damage, reflecting the delay in their presentation and diagnosis. Adherence to GFD remains a problem; therefore, a thorough assessment and counseling at the time of diagnosis and ongoing care are crucial.
